# Association between tobacco-related social environment and e-cigarette use among young adults in northern Vietnam: A cross-sectional study with latent class analysis

**DOI:** 10.18332/tid/218632

**Published:** 2026-03-31

**Authors:** Manh Cuong Vu, Wanrudee Isaranuwatchai, Rassamee Chotipanvithayakul, Wit Wichaidit

**Affiliations:** 1 Department of Epidemiology, Faculty of Medicine, Prince of Songkla University, Hat Yai, Thailand; 2 Health Strategy and Policy Institute, Hanoi, Vietnam; 3 Health Intervention and Technology Assessment Program Foundation, Nonthaburi, Thailand

**Keywords:** tobacco use, social environment, e-cigarette use, young adults, Vietnam

## Abstract

**INTRODUCTION:**

Tobacco and e-cigarette use among cohabitants and friends tends to cluster together, but data are scarce regarding the association between the clustering patterns and e-cigarette use. The objectives of this study are to identify clusters of the tobacco-related environment and assess the extent to which these clusters are associated with e-cigarette use among young adults in Vietnam.

**METHODS:**

We carried out a cross-sectional study among Vietnamese undergraduate students in the second year of study or higher at two universities in Hanoi between September and December 2024. We used latent class analysis to identify clusters in a tobacco-related social environment. We performed multivariable logistic regression to assess the association between these clusters and ever e-cigarette use.

**RESULTS:**

Among our participants (n=1448 students), three distinct clusters/classes emerged: 1) Low exposure – participants with a low level of exposure to tobacco use in the social environment; 2) Parent smokers – participants who had parents who currently smoked; and 3) Peer smokers and/or e-cigarette users – participants with friends who currently smoked cigarettes and/or used e-cigarettes (64.9%, 24.6% and 11.5% of all participants, respectively). Participants from the parent smokers class (adjusted odds ratio, AOR=2.15; 95% CI: 1.06–4.34) and the peer smokers and/or e-cigarette users class (AOR=6.33; 95% CI: 3.10–13.07) were significantly more likely to be e-cigarette ever users than those from the low exposure class.

**CONCLUSIONS:**

The most common smokers in the social environment were parents and peers, whilst the most typical e-cigarette users were peers. The influence of having peers who smoked cigarettes and/or used e-cigarettes was stronger than the influence of having parents who smoked. The study findings provide basic information that may be useful to stakeholders in designing tailored anti-e-cigarette use campaigns. Campaigns should consider focusing on norms and spaces where young people often socialize.

## INTRODUCTION

E-cigarettes are electronic chemical delivery systems that heat a liquid (e-liquid) to create aerosols inhaled by the users^1^. E-liquids possess additives, flavors, and other substances that can harm people’s health^1^. E-cigarettes commonly have nicotine content, and several types possess nicotine salts, which enable users to inhale high levels of nicotine in the absence of common adverse effects found in conventional cigarettes^2^. E-cigarette use has grown swiftly despite the relatively recent emergence in the market^3^. It is estimated that there are approximately 101 million e-cigarette users worldwide, including 86 million adults and 15 million adolescents^4^. In the World Health Organization’s Western Pacific Region, around 22 million people use e-cigarettes, including approximately 5 million adolescents^4^.

Considering the high number of users and harm of e-cigarettes, data are currently needed regarding behavioral determinants. A published systematic review has identified both individual-level and interpersonal risk factors^5^. Considering the social-ecological model of health behaviors^6^, the extent to which the social environment influences e-cigarette use warrants further exploration. The social environment is defined as the social conditions that affect one’s living conditions and behaviors^7^. The social environment also includes health behaviors, such as tobacco use, among one’s family members and peers, that influence one’s own smoking and e-cigarette use^5,8^. However, health behaviors of family members and peers tend to cluster into patterns^9-12^. Latent class analysis is an analysis technique for categorizing individuals into mutually exclusive clusters^13^. This technique can be applied to classifying individuals according to the characteristics of their social environment. Thus, latent class analysis results can provide insights into how tobacco use among family members and peers influences e-cigarette use.

Vietnam is a low-middle-income country ranked among the top 5 countries in the World Health Organization’s Western Pacific Region concerning the number of tobacco smokers (approximately 16 million persons)^4^. In Vietnam, 4.6% of general adults have ever used e-cigarettes^14^, and the value is as high as 7.4% among young adults in metropolitan cities^15^. Previous studies in Vietnam analyzed the influence of each person in circles of family and friends separately^14,16^. However, the social environment exists as patterns and likely exerts influence collectively rather than as individual, independent units. This study aimed to describe the patterns of tobacco use in the social environment among young adults in northern Vietnam and the extent to which these patterns are associated with e-cigarette use.

## METHODS

### Study design and setting

We conducted a cross-sectional study in Hanoi, Vietnam, between September and December 2024.

### Study participants and sample size calculation

The study participants were Vietnamese undergraduate students aged 18–30 years. We recruited students in their second year or higher level to reduce the probability of encountering students under 18 years of age who would be ineligible. This study was a part of a survey that described current e-cigarette use by young adults. We hypothesized that 13.2% of students are current e-cigarette users16. At a 95% confidence level, a 3.5% arbitrary margin of error, and a design effect of 2, we obtained a sample size of 719 students per university. We assumed an arbitrary 10% non-response rate and adjusted the sample size to 800 students per university, for a total of 1600 students.

We used a multistage clustered sampling to select our participants. We randomly selected one public medical university and one public non-medical university for the diversity of study participants. Next, we randomly selected classes at each university and invited all eligible students to participate until reaching the required sample size.

The study was approved by the Institutional Review Board of the Health Strategy and Policy Institute, Vietnam (approval number: 245/QD-CLCSYT). Participants provided informed consent.

### Study instrument

The study instrument was a self-administered questionnaire. We designed the questionnaire in English and adapted it from published and validated instruments^17-19^. We then translated the questionnaire into Vietnamese, and another Vietnamese health professional back-translated the questionnaire into English. We compared these versions and corrected them in the corresponding parts of the Vietnamese translation. We consulted Vietnamese public health practitioners and e-cigarette users regarding the content of the drafted questionnaire. We then conducted a pilot test of the questionnaire among Vietnamese young adults to evaluate its phrasing and structure, and finalized the questionnaire for data collection. All of them found the questionnaire easy to understand and answer.

### Study variables

*Exposure: tobacco-related social environment*

We measured the tobacco-related social environment using two questions. The questions were: ‘Do any of the people living with you or your friends smoke conventional cigarettes now?’ and ‘Do any of the people living with you or your friends use e-cigarettes now?’. We categorized cohabitants and friends into several groups, including grandparents, parents, siblings, friends, cousins/brothers-in-law, and uncles/ aunts/relatives.

*Outcome: e-cigarette use*

We measured e-cigarette use with the question: ‘Have you ever used e-cigarettes (even if just a few puffs)?’. Participants who answered ‘Yes’ were considered to be ever users of e-cigarettes.

*Characteristics of the study participants*

We included questions regarding the participants’ age, sex, ethnicity, campus location, and place of residence. We also asked participants about their history of smoking with the question: ‘Have you ever smoked conventional cigarettes (even if just a few puffs)?’.

### Data collection

We contacted the sampled universities to conduct the study. We visited the selected universities to inform the administrative staff about the study. We also discussed methods for approaching students in a context-suitable manner and reached a consensus on the matter. We then asked the university’s student volunteers to assist in distributing the participant information sheet and consent forms to students and to invite them to participate in the study. The participants were informed about the study objectives, the confidentiality of their identities, the voluntary nature of their participation, and their right to skip any question. Once informed consent had been secured, students proceeded to complete either the online questionnaire or the paper-and-pen questionnaire if they were unable to access the online form.

### Data analyses

We stored data in an encrypted, password-protected system and routinely cleaned the data. We analyzed the study data using descriptive statistics to summarize the prevalence of the tobacco-related social environment. For categorical variables, we used frequencies and percentages, and either the chi-squared or Fisher’s exact test. For continuous variables, we used parametric or nonparametric tests depending on the distribution’s normality.

We then used latent class analysis to identify patterns of tobacco use within the social environment. Based on responses to the two social environment questions, we derived a total of 12 dummy variables. Using these dummy variables, we constructed latent class models with increasing numbers of latent classes. We then considered the Akaike information criterion, Bayesian information criterion, and sample size-adjusted Bayesian information criterion to balance model complexity against sample size, the maximum log-likelihood statistic for identifying the values most likely to have produced the observed data, and entropy for classification quality. We cross-tabulated the latent classes in each output against tobacco use in the social environment to validate the findings. Afterward, we deliberated on the model fit parameters and the cross-tabulation outputs to choose the output that yielded the most meaningful clusters and assigned names to the latent classes.

We cross-tabulated the latent classes outputs with the participants’ e-cigarette use behavior and used unadjusted logistic regression for the bivariable analyses. We then performed multivariable logistic regression models to adjust for the effects of the participants’ age, sex, ethnicity, campus location, place of residence, and smoking history. Previous studies have identified these characteristics as independent predictors of the outcome^14,16,20-22^. Thus, we consider them confounders in our analyses. We performed all statistical analyses using R version 4.5.1^23^ at the 95% confidence level.

## RESULTS

### Prevalence of tobacco-related social environment

Among our 1448 participants, the majority reported low to no exposure to tobacco use in their social environment. The most commonly-reported smokers in the participants’ social environment were parents (reported by 27.7% of all participants) and friends (16.5%). The participants’ friends were the sole commonly reported group of e-cigarette users (20.9%) (Table 1).

**Table 1:** Distribution of smoking and e-cigarette use in the participants’ social environment, cross-sectional study, Hanoi, Vietnam, 2024 (N=1448)

*Social environment*	*Smoking n (%)*	*Vaping n (%)*
Grandparent	76 (5.6)	5 (0.4)
Parent	376 (27.7)	10 (0.8)
Sibling	82 (6.0)	25 (1.9)
Friend	224 (16.5)	274 (20.9)
Cousin/brother-in-law	8 (0.6)	4 (0.3)
Uncle/aunt/relative	31 (2.3)	1 (0.1)
No one	714 (52.6)	998 (76.1)

### Latent class model

The three-class latent class model showed the highest fit for specific indicators (Table 2). The model with three classes had the lowest sample size-adjusted Bayesian information criterion (SSABIC) value, indicating the best fit. Further validation with cross-tabulation also confirmed the suggestion. We then reached a consensus that the three classes could distinguish the participants in the most parsimonious manner and used the outputs for further analysis.

**Table 2:** Fit indices for the latent class model for tobacco-related social environment, cross-sectional study, Hanoi, Vietnam, 2024

*Index*	*1 class*	*2 classes*	*3 classes*	*4 classes*	*5 classes*	*6 classes*
AIC	5836	5549	5490	5470	5467	5477
BIC	5898	5678	5686	5733	5797	5874
SSABIC	5860	5599	5565	5571	5593	5629
Max. LL	-2906	-2749	-2707	-2684	-2669	-2661
Entropy	NC	1	0.714	0.846	0.767	NC

AIC: Akaike information criterion. BIC: Bayesian information criterion. SSABIC: Sample size-adjusted Bayesian information criterion. Max. LL: maximum log-likelihood statistic. NC: not computable.

Among the three latent classes (Table 3), the majority of participants (64.9%) belonged to Class 1. Participants in Class 1 included those who had few to no people in their social environment who smoked or used e-cigarettes. Thus, we labeled this class the low exposure group. Class 2 included approximately one-quarter of the participants, and nearly all participants in this class lived with a parent who smoked, so we named this class the parent smokers group. The rest of the participants were in Class 3, where everyone had friends who smoked and/or used e-cigarettes, so we referred to this class as the peer smokers and/or e-cigarette users group.

**Table 3:** Distribution of tobacco-related social environment by latent class, cross-sectional study, Hanoi, Vietnam, 2024 (N=1273)

*Behavior*	*Class 1* *Low exposure* *(N=826)* *n (%)*	*Class 2* *Parent smokers* *(N=300)* *n (%)*	*Class 3* *Peer smokers and/or e-cigarette users* *(N=147)* *n (%)*
**Smoking**			
Grandparent	32 (3.9)	25 (8.3)	10 (6.8)
Parent	0 (0)	289 (96.3)	46 (31.3)
Sibling	25 (3.0)	37 (12.3)	13 (8.8)
Friend	59 (7.1)	10 (3.3)	147 (100)
Cousin/brother-in-law	2 (0.2)	1 (0.3)	1 (0.7)
Uncle/aunt/relative	21 (2.5)	0 (0)	3 (2.0)
**E-cigarette use**			
Grandparent	0 (0)	3 (1.0)	2 (1.4)
Parent	0 (0)	7 (2.3)	3 (2.0)
Sibling	0 (0)	16 (5.3)	8 (5.4)
Friend	68 (8.2)	52 (17.3)	147 (100)
Cousin/brother-in-law	4 (0.5)	0 (0)	0 (0)
Uncle/aunt/relative	1 (0.1)	0 (0)	0 (0)

### Differences between classes

Participants in all three classes were primarily female and were on an urban campus (Table 4). The one notable distinction seemed to be in the history of smoking, which was notably more common in the peer smokers and/or e-cigarette users group than in other groups. The other characteristics (age, ethnicity, and place of residence) appeared to be similar.

**Table 4:** Distribution of participant’s demographic and socio-economic characteristics by latent classes, cross-sectional study, Hanoi, Vietnam, 2024 (N=1273)

*Characteristics*	*Low exposure* *(N=826)* *n (%)*	*Parent smokers* *(N=300)* *n (%)*	*Peer smokers and/or* *e-cigarette users* *(N=147)* *n (%)*	*p*
**Sex**				
Male	279 (33.9)	81 (27.1)	60 (41.1)	0.009
Female	545 (66.1)	218 (72.9)	86 (58.9)	
**Age** (years)				
Median (IQR)	20 (20–21)	20 (20–21)	20 (20–21)	0.335
**Ethnicity**				
Kinh	712 (86.5)	269 (90.6)	127 (87.0)	0.188
Other	111 (13.5)	28 (9.4)	19 (13.0)	
**Campus location**				
Urban	429 (51.9)	187 (62.3)	80 (54.4)	0.008
Peri-urban	397 (48.1)	113 (37.7)	67 (45.6)	
**Place of residence**				
Private house	188 (23.0)	78 (26.1)	21 (14.3)	0.116
Relative’s house	32 (3.9)	15 (5.0)	5 (3.4)	
Rental house	457 (55.9)	164 (54.8)	94 (63.9)	
Dormitory	140 (17.1)	42 (14.0)	27 (18.4)	
**History of smoking**				
Ever smoked	48 (5.9)	26 (8.7)	30 (20.4)	<0.001
Never smoked	769 (94.1)	273 (91.3)	117 (79.6)	

IQR: interquartile range.

### Association between latent class and ever e-cigarette use

We found significant associations between latent classes of exposure to tobacco use in the social environment and ever e-cigarette use (Table 5). Participants in the parent smokers group had twice as high ever e-cigarette use as participants in the low exposure group (7.7% vs 4.0%; AOR=2.15; 95% CI: 1.06–4.34). Participants in the peer smokers and/or e-cigarette users group had up to six times higher ever e-cigarette use compared to participants in the low exposure group (23.3% vs 4.0%; AOR=6.33; 95% CI: 3.10–13.07).

**Table 5:** Association between latent class and ever e-cigarette use, cross-sectional study, Hanoi, Vietnam, 2024 (N=1245)

*Latent class*	*Ever e-cigarette use*		*OR (95% CI)*	*AOR (95% CI)*
	*No* *n (%)*	*Yes* *n (%)*		
Low exposure (ref.)	788 (96.0)	33 (4.0)	1	1
Parent smokers	275 (92.3)	3 (7.7)	**2.00 (1.14–3.44)**	**2.15 (1.06–4.34)**
Peer smokers and/or e-cigarette users	112 (76.7)	34 (23.3)	**7.25 (4.31–12.21)**	**6.33 (3.10–13.07)**

AOR: adjusted odds ratio; adjusted for sex, age, ethnicity, campus location, place of residence, and history of smoking.

## DISCUSSION

In this study, we identified three patterns of exposure to tobacco use in the social environment among university-attending young people in Hanoi, Vietnam. We found that numerous patterns of exposure to tobacco use had various levels of association with ever e-cigarette use, and the findings suggested that the influence of tobacco use among peers on one’s own e-cigarette use was strongest, followed by the influence of tobacco use by one’s parents. Our study’s findings may have implications for tobacco-use prevention programs among young people.

Latent class analysis yielded three distinct patterns of tobacco use in the social environment. Aside from the majority in the low exposure group, there was a clear distinction between participants in the parent smokers group, who rarely had peers who used tobacco, and participants in the peer smokers and/or e-cigarette users group, who all had peers who smoked and also had friends who used e-cigarettes. The strong correlation between having peers who smoked conventional cigarettes and peers who used e-cigarettes could be attributed to the group members’ own sense of identity, according to the social identity theory^24^. However, information gaps existed in our measurement. We asked about the current use of conventional cigarettes and e-cigarettes among various groups in the social environment, not the frequency or amount of use, history of use, or the extent of influence members of each group had on the participants, which did not allow for complete interpretation of the variations in these patterns and e-cigarette use behaviors as per theories of social norms^25^. Future studies should specify that participants report only on their significant others and quantify the median frequency and amount of conventional cigarette and e-cigarette use.

We found that young people who had friends who smoked and/or used e-cigarettes had a higher likelihood of e-cigarette use themselves than those who only had parents who smoked, which was higher than the likelihood among those who had low to no exposure. The influence of peer and parental tobacco use on one’s own behavior has been documented in the literature^20,26,27^. The more substantial influence of peers over parents may reflect the inherent nature of social dynamics among the young. Young people may observe friends using e-cigarettes, perceive favorable reactions to e-cigarettes, become inquisitive about unexplored experiences, and then mimic and model behavior based on social learning theory^28^. This issue is widespread among those who have completed general schooling and aspire to autonomy from their parents, living in an environment that fosters interactions with friends^29^. In addition, e-cigarette users may develop a supportive e-cigarette-related environment, which uplifts others in the network to begin using e-cigarettes^30^. Proverbs such as *‘Chọn bạn mà chơi’* (Literal translation: ‘Choose your friends wisely’) serve as examples of the inherent influence of social norms from peers that may not be evident in observing deviancy among parents, similar to Peer Cluster Theory^31^. Our study’s findings provide basic information that is potentially useful for stakeholders in designing tailored anti-e-cigarette use campaigns, which should consider focusing on norms and spaces where young people often socialize.

### Strengths and limitations

The strength of our study was the inclusion of extended families in the reporting of both smoking and e-cigarette use in the social environment, which enabled a comprehensive assessment of the behavior. However, several limitations should be considered when interpreting our study findings. Firstly, the cross-sectional study design precluded the establishment of causal relationships. Next, tobacco use is considered a deviant behavior in Vietnamese society. Thus, social desirability could have influenced the (under)reporting of the exposure and/or the outcome. In addition, our participants consisted only of undergraduate students from two universities with a bigger proportion of females, which may not be generalizable to young adults in Vietnam in other settings. In addition, we did not measure the exposure to smoking and e-cigarette use by social media influencers, which could have confounded the observed associations. Future studies should consider making such measurements to provide a more accurate assessment of the associations accordingly. Lastly, our study participants might have been reluctant to self-report tobacco and e-cigarette use. Thus, social desirability bias should not be ruled out when interpreting our study findings.

## CONCLUSIONS

Our study found three classes of tobacco-related social environment among Vietnamese young adults: 1) Low exposure, 2) Parent smokers, and 3) Peer smokers and/or e-cigarette users. Participants in the peer smokers and/or e-cigarette users group were significantly more prone to use e-cigarettes compared to the parent smokers group and the low exposure group. The study findings provided potentially useful basic information for stakeholders in designing tailored e-cigarette prevention campaigns, focusing on messages centered on norms and on spaces where young people often socialize. Future studies should explore in detail the volume of cigarette smoking and e-cigarette use, the closeness of the relationship among young people and their cohabitants and peers, as well as the impact of their favorite social media influencers.

## Data Availability

The data supporting this research are available from the authors upon reasonable request.
